# CRMP2 is necessary for Neurofibromatosis type 1 related pain

**DOI:** 10.1080/19336950.2017.1370524

**Published:** 2017-08-24

**Authors:** Aubin Moutal, Song Cai, Shizhen Luo, Raphaëlle Voisin, Rajesh Khanna

**Affiliations:** a Department of Pharmacology, University of Arizona, Tucson, AZ, USA; b Department of Anesthesiology, University of Arizona, Tucson, AZ, USA; c Neuroscience Graduate Interdisciplinary Program, College of Medicine, University of Arizona, Tucson, AZ, USA

**Keywords:** CaV2.2, CRISPR/Cas9, naV1.7, neurofibromatosis, neuropathy

## Abstract

Neurofibromatosis type 1 (NF1) is one of the most common genetic diseases, affecting roughly 1 in 3000 individuals. As a multisystem disorder, it affects cognitive development, as well as bone, nerve and muscle constitution. Peripheral neuropathy in NF1 constitutes a potentially severe clinical complication and is associated with increased morbidity and mortality. The discovery of effective therapies for Neurofibromatosis type 1 (NF1) pain depends on mechanistic understanding that has been limited, in part, by the relative lack of availability of animal models relevant to NF1 pain. We have used intrathecal targeted editing of *Nf1* in rats to provide direct evidence of a causal relationship between neurofibromin and pain responses. We demonstrated that editing of neurofibromin results in functional remodeling of peripheral nociceptors characterized by enhancement of interactions of the tetrodotoxin-sensitive (TTX-S) Na^+^ voltage-gated sodium channel (NaV1.7) and the collapsin response mediator protein 2 (CRMP2). Collectively, these peripheral adaptations increase sensory neuron excitability and release of excitatory transmitters to the spinal dorsal horn to establish and maintain a state of central sensitization reflected by hyperalgesia to mechanical stimulation of the hindpaw. The data presented here shows that CRMP2 inhibition is sufficient to reverse the dysregulations of voltage-gated ion channels and neurotransmitter release observed after *Nf1* gene editing. The concordance in normalization of ion channel dysregulation by a CRMP2-directed strategy and of hyperalgesia supports the translational targeting of CRMP2 to curb NF1-related pain.

Neurofibromatosis type 1 (NF1) is the most commonly inherited neurological human disorder, affecting about 1 in 3000 people worldwide and is caused by heterozygous mutations of the *Nf1* gene.^1^ More than 1000 different mutations that result in expression of a mutated or truncated neurofibromin protein have been reported^1^. Neurofibromin, the protein coding the *Nf1* gene, is mostly expressed in the nervous system and is implicated in neuronal differentiation^2^ and learning^3^. Inactivating mutations of the *Nf1* gene result in unchecked Ras (small GTPase acting as pro-oncogene) activation leading to tumorigenesis; seizures; cognitive disorders and pain.^1^ A recent study of 531 NF1 patients identified a significant burden of autistic traits and symptoms in this monogenic syndrome. NF1 patients have a 50% incidence of deficits in social behavior.^4^ Of these individuals, 30–52% of cases will be severe enough to be clinically diagnosed with an autism spectrum disorder (ASD).^5-7^ Unchecked GABAergic input has been hypothesized to account for the learning and social deficits in NF1 patients.^3,8,9^ Recently, heterozygous loss of *Nf1* was found to increase the activity of interneurons because of decreased hyperpolarization-activated cyclic nucleotide-gated channel (HCN) function, resulting in increased inhibitory inputs and decreased memory formation.^8^

Although inhibitory interneurons were found to be responsible for NF1 related learning and social deficits, other neurons might be affected and contribute to different symptoms of the disease.^9^ Another major clinical feature of NF1 is chronic idiopathic pain.^10,11^ The occurrence of neuropathic symptoms in NF1 adds to the overall neurologic disability of patients and is associated with increased morbidity and mortality. While this aspect of the disease has been often overlooked, a recent study used clustered regularly interspaced short palindromic repeats (CRISPR) associated protein-9 nuclease (Cas9) gene editing of *Nf1* in rats to show a direct link between the expression of a truncated neurofibromin and the development of hyperalgesia^12^. These nociceptive behaviors were caused by a loss of neurofibromin interaction with the Collapsin Response Mediator Protein 2 (CRMP2), a protein involved in pain signal transmission and dysregulated in neuropathic pain.^13^ CRMP2 controls the release of calcitonin related gene peptide (CGRP), a neurotransmitter for nociceptive signals, through its interaction with Syntaxin 1A.^14^ Under loss of neurofibromin expression, CRMP2 interaction with syntaxin 1A was increased.^14^ This suggested that the loss or truncation of neurofibromin in NF1 results in increased CGRP release and hyperalgesia through CRMP2.

To investigate the role of CRMP2 in NF1-related pain, we constructed a CRISPR/Cas9 lentiviral approach for *Nf1* gene editing in adult rats by inserting the gRNA sequence (GGCAGTAACCCTTTGTCGTT, score 86) targeting exon 39, into the plasmid pL-CRISPR.EFS.tRFP (Cat# 57819, Addgene).^15^ Lentiviral particles were generated (Viracore, UCSF) and 5 × 10^5^ (in 15µl) live lentiviruses were injected into the lumbar region of the spinal cord through an intrathecal catheter. All sensory neurons from the lumbar dorsal root ganglia (DRG, L4, L5, L6) were transduced (Fig. 1a, panel tRFP). Using antibodies specific against the N- or the C-terminus of neurofibromin, we detected efficient genome editing the *Nf1* gene resulting in the expression of a truncated neurofibromin (Fig. 1a). We next tested if the release of the nociceptive neurotransmitter CGRP was altered in *Nf1* edited animals. CGRP release was evoked from the lumbar regions of the spinal cord by depolarization with a Tyrode's solution containing 90 mM KCl (as before^14^). *Nf1* gene editing lead to an increase of evoked CGRP release by 94% compared to control animals (injected with a virus containing no gRNA sequence) (Fig. 1b). As neurofibromin's interaction with CRMP2 limits CRMP2s interaction with syntaxin 1A and CGRP release,^14^ we hypothesized that CRMP2 was the ‘master regulator’ underlying NF1-related pain. CRMP2 regulates the neuronal voltage gated Na^+^ channels whose currents are increased in NF1. We then knocked-down CRMP2 using an siRNA (validated in ^16,17^) in *Nf1* edited DRGs to reverse the increase of Na^+^ currents described before.^12^ *Nf1* gene editing using our novel lentivirus resulted in increased Na^+^ currents (Fig. 1c, d). CRMP2 interference normalized the Na^+^ currents to the level of the control (no gRNA virus). These results show that CRMP2 is necessary for increasing Na^+^ currents downstream of *Nf1* gene editing.

**Figure 1.**
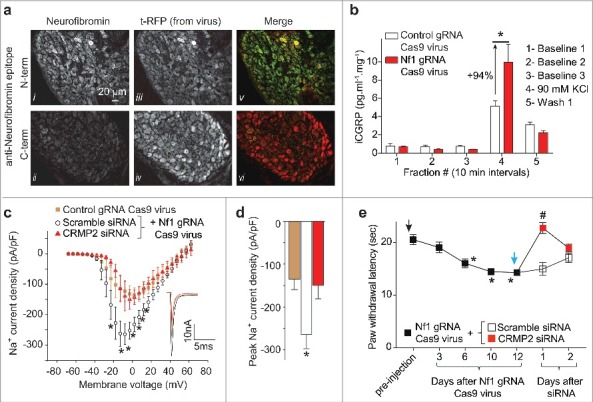
Cas9-mediated *Nf1* gene editing increases CRMP2-dependent CGRP release, Na^+^ currents and hyperalgesia. (a) Representative micrographs of a 10-μm section of adult dorsal root ganglia (DRG) from rats 10 days after an intrathecal injection of lentivirus (5 × 10^5^ in 15µl) expressing both *Cas9* and a gRNA targeting exon 39 of *Nf1* and immunostained with neurofibromin (*i*, *ii*), t-RFP shows in vivo viral transduction in DRG (*iii*, *iv*). The merge panel shows N-terminus neurofibromin signal (*v*) and loss of C-terminus neurofibromin (*vi*) signal in transduced DRGs. Scale bar: 20 µm. (b) Spinal cords from adult rats injected with control or *Nf1* gRNA lentivirus particles were used to assess potassium chloride (KCl, 90 mM)-induced CGRP release from nerve terminals. KCl increased CGRP release in spinal cords in *Nf1* gRNA lentivirus-injected rats, which was significantly higher than in cords from control gRNA lentivirus-injected rats (* p < 0.05 vs. control; two-way ANOVA *post hoc* Sidak's test). Y-axis shows immunoreactive CGRP levels in the bath solution and normalized to the weight of each spinal cord. (c) Current-voltage relationship of tetrodotoxin-sensitive (TTX-S) Na^+^ currents from sensory neurons transfected with lentiplasmid containing either control or Nf1 sgRNAs in additional presence of scramble or of CRMP2 siRNA (n = 9–10 per condition). *Inset* shows peak traces from the indicated conditions. (d) Peak current densities at –10 mV for the indicated conditions. (e) Male rats injected with Nf1 sgRNA lentiviral particles (*black arrow*) show a behavioral deficit in response to the radiant heat in the Hargreaves test in comparison to uninjected rats (n = 12; (*P < 0.05 vs. pre-injection baseline, 2-way ANOVA, post hoc Student-Neuman-Kuels). Thermal hyperalgesia was completely blocked by injection (*blue arrow*), at day 12, of CRMP2, but not scramble, siRNA (n = 12; ^#^*P* < 0.05 vs. pre-injection baseline, 2-way ANOVA, post hoc Student-Neuman-Kuels). The Institutional Animal Care and Use Committee of the College of Medicine at the University of Arizona approved all experiments. All behavioral experiments were performed by experimenters who were blinded to randomly assigned experimental groups and treatments. Male Sprague–Dawley rats (225 g, Envigo, Placentia, CA) were used for all studies.

Increased CGRP release and Na^+^ currents suggests that *Nf1* gene edited animals will develop nociceptive behaviors in a CRMP2-dependent manner. To test this, we injected rats with the *Nf1* gRNA Cas9 lentivirus. Over the course of 10 days, rats developed hyperalgesia measured by decreased paw withdrawal latency (Hargreaves' method). At day 12 after viral injection, rats were injected (i.t.) with CRMP2 or scramble siRNA^17^ complexed to TurboFect *in vivo* (Cat# R0541, ThermoFisher) for *in vivo* siRNA transfection. At 24 hours after injection of CRMP2 siRNA, nociceptive behaviors were reversed in Nf1 edited rats (Fig. 1e). That hyperalgesia returned at 48 hours is consistent with the turnover of CRMP2 over this period^18^ and a limitation of the non-viral transfection method used here. Scramble siRNA injected rats did not show any change in their paw withdrawal threshold (Fig. 1e). This data shows that NF1 related pain involves CRMP2 as a central node and genetic targeting of CRMP2 is sufficient to alleviate painful behaviors.

The advantage of the spinal CRISPR/Cas9 approach used here is that it allows us to model a potential mechanism relevant to a symptom experienced by NF1 patients, rather than attempting to capture the full spectrum of the disease itself. Importantly, anxiety-related behaviors in male and female rats were not affected by intrathecal *Nf1* editing,^12^ demonstrating that our reductionist approach of symptom-specific modeling of a multi-faceted disease may permit mechanistic studies into NF1 pain as well as other neurological symptoms.

The results presented here are an improvement upon the published method for *in vivo* Nf1 gene editing^12^ by reducing the protocol to one single lentivirus injection. Published research on NF1 focuses on the involvement of the loss of the Ras inactivating domain of NF1 as being responsible for all of the patients' symptoms. Our strategy spares the Ras inactivating domain but recapitulates a major symptom affecting the patients' quality of life and comorbidity factor, chronic idiopathic pain. We found that the loss of neurofibromin regulation of CRMP2 was responsible for NF1-related pain.^14^ The data presented here shows that CRMP2 inhibition is sufficient to reverse the dysregulations of voltage-gated ion channels and neurotransmitter release observed after *Nf1* gene editing. The concordance in normalization of ion channel dysregulation by a CRMP2-directed strategy and of hyperalgesia supports the translational targeting of CRMP2 to curb NF1-related pain.
